# Ribonuclease H/DNA Polymerase HIV-1 Reverse Transcriptase Dual Inhibitor: Mechanistic Studies on the Allosteric Mode of Action of Isatin-Based Compound RMNC6

**DOI:** 10.1371/journal.pone.0147225

**Published:** 2016-01-22

**Authors:** Angela Corona, Rita Meleddu, Francesca Esposito, Simona Distinto, Giulia Bianco, Takashi Masaoka, Elias Maccioni, Luis Menéndez-Arias, Stefano Alcaro, Stuart F. J. Le Grice, Enzo Tramontano

**Affiliations:** 1 Department of Life and Environmental Sciences, University of Cagliari, Cagliari, Italy; 2 National Cancer Institute, Frederick, Maryland, United States of America; 3 Centro de Biología Molecular “Severo Ochoa” (Consejo Superior de Investigaciones Científicas & Universidad Autónoma de Madrid), Madrid, Spain; 4 Dipartimento di Scienze della Salute, Università Magna Graecia di Catanzaro, Campus “S. Venuta”, Viale Europa, 88100, Catanzaro, Italy; 5 Istituto di Ricerca Genetica e Biomedica, Consiglio Nazionale delle Ricerche (CNR), Monserrato, Cagliari, Italy; Meharry Medical College, UNITED STATES

## Abstract

The DNA polymerase and ribonuclease H (RNase H) activities of human immunodeficiency virus type 1 (HIV-1) are needed for the replication of the viral genome and are validated drug targets. However, there are no approved drugs inhibiting RNase H and the efficiency of DNA polymerase inhibitors can be diminished by the presence of drug resistance mutations. In this context, drugs inhibiting both activities could represent a significant advance towards better anti-HIV therapies. We report on the mechanisms of allosteric inhibition of a newly synthesized isatin-based compound designated as RMNC6 that showed IC_50_ values of 1.4 and 9.8 μM on HIV-1 RT-associated RNase H and polymerase activities, respectively. Blind docking studies predict that RMNC6 could bind two different pockets in the RT: one in the DNA polymerase domain (partially overlapping the non-nucleoside RT inhibitor [NNRTI] binding pocket), and a second one close to the RNase H active site. Enzymatic studies showed that RMNC6 interferes with efavirenz (an approved NNRTI) in its binding to the RT polymerase domain, although NNRTI resistance-associated mutations such as K103N, Y181C and Y188L had a minor impact on RT susceptibility to RMNC6. In addition, despite being naturally resistant to NNRTIs, the polymerase activity of HIV-1 group O RT was efficiently inhibited by RMNC6. The compound was also an inhibitor of the RNase H activity of wild-type HIV-1 group O RT, although we observed a 6.5-fold increase in the IC_50_ in comparison with the prototypic HIV-1 group M subtype B enzyme. Mutagenesis studies showed that RT RNase H domain residues Asn474 and Tyr501, and in a lesser extent Ala502 and Ala508, are critical for RMNC6 inhibition of the endonuclease activity of the RT, without affecting its DNA polymerization activity. Our results show that RMNC6 acts as a dual inhibitor with allosteric sites in the DNA polymerase and the RNase H domains of HIV-1 RT.

## Introduction

Since the identification of the human immunodeficiency virus (HIV) as a retrovirus causing AIDS [1, 2], it was clear that the viral reverse transcriptase (RT) was an excellent target for drug intervention. During reverse transcription the (+) single-stranded viral genomic RNA is converted to a particular integration-competent double-stranded viral DNA, in a process that is entirely catalyzed by the RT. HIV type 1 (HIV-1) RT is a multifunctional heterodimeric enzyme composed of subunits of 66 and 51 kDa (p66/p51), with DNA polymerase and ribonuclease H (RNase H) activities. For DNA polymerization, RTs can use as templates either RNA (RNA-dependent DNA polymerase (RDDP)) or DNA (DNA-dependent DNA polymerase (DDDP)). DNA polymerase and RNase H activities are both essential for viral replication [3], and are located in two separated domains of the p66 RT subunit. The DNA polymerase domain is located at the N-terminus and exhibits the classical “right hand” conformation, while the RNase H domain is located at the C-terminus, 60 Å away from the polymerase active site. The distance between the active sites of the polymerase and the RNase H is estimated at around 17–18 base pairs, and both domains are linked by a so-called connection subdomain. Long-range effects and functional interdependence between active domains are been suggested [4, 5], based on mutational studies showing that residues such as Pro226, Phe227, Gly231, Tyr232, Glu233, and His235 at the polymerase domain of the HIV-1 RT could affect RNase H activity [6], whereas deletions at the C-terminus can decrease the efficiency of DNA polymerization [7]. Such structural and functional interdependence is also supported by evidence showing that mutations in the RNase H domain could affect resistance to nucleoside RT inhibitors (NRTIs) [6, 8–10], while NNRTIs such as nevirapine and efavirenz (EFV) increase RNase H activity upong binding HIV-1 RT [11, 12].

Because of their pivotal role in viral replication, RDDP and RNase H activities are both validated targets for the identification of new RT inhibitors, needed to combat the emergence of multi-drug resistant strains, whose spreading in newly infected patients is an issue of increasing concern, causing a number of associated antiviral therapy failures [13]. In this scenario, the identification of a compound with the ability to inhibit both activities could represent a significant advance in the fight against drug resistance and could reduce the number of pills and the dose of administered drugs [14]. Drugs targeting the DNA polymerase activity (i.e. RDDP inhibitors, and DDDP inhibitors) acting on nucleotide incorporation (i.e. NRTIs) or allosteric drugs (i.e. NNRTIs), are commonly used in current therapies. On the contrary, RNase H activity is a more challenging target with no drugs available for clinical use, although three classes of molecules have shown inhibitory activity in preclinical studies [15–17]: i) metal-chelating active site inhibitors, ii) allosteric p66/p51 interface inhibitors, and iii) allosteric RDDP RNase H dual inhibitors. Several compounds of the last group have been identified as inhibitors of both RT functions *in vitro* [18]. In particular, hydrazones have been reported to inhibit RNase H function by accessing through an allosteric pocket located in the vicinity of the NNRTI binding site [19]. More recently, a second hydrazone binding site, located at the RNase H domain, has been proposed [20–22].

**Fig 1:**
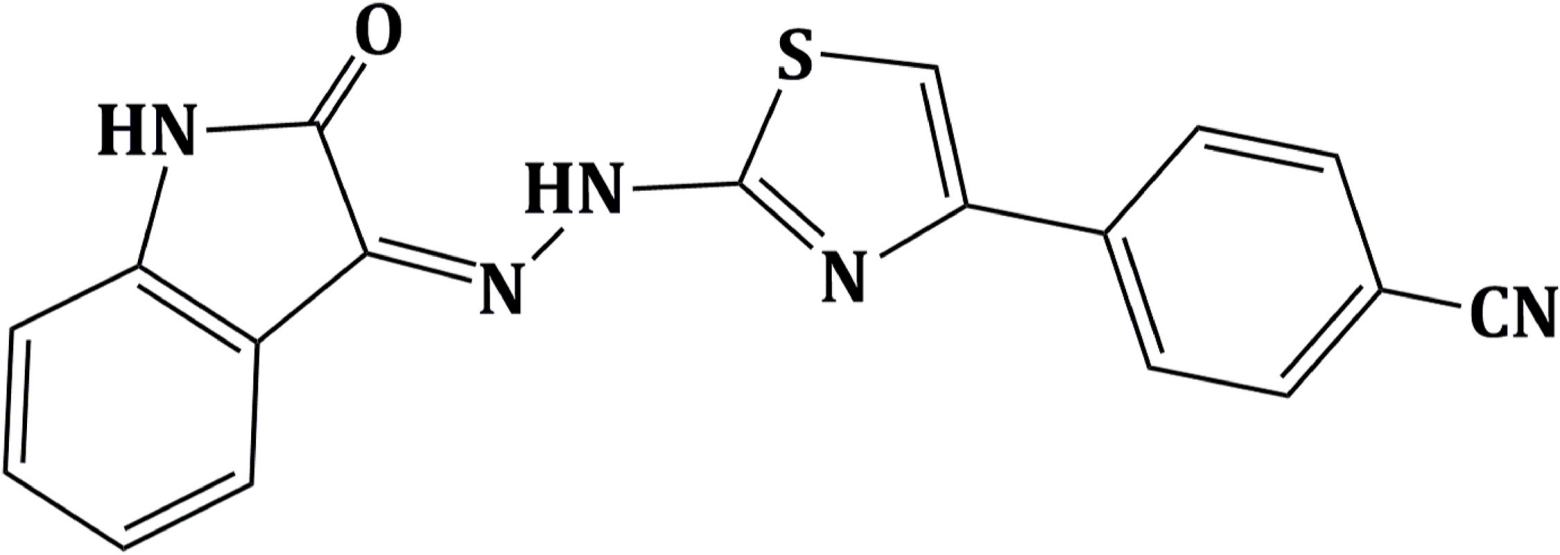
Chemical structure of the isatin derivative RMNC6.

With the aim of identifying new HIV-1 RNase H inhibitors, we previously performed a successful ligand-based virtual screening identifying hydrazoindolin-2-one derivatives, active against RNase H and DNA polymerase functions of the RT, in the low micromolar range [23]. This scaffold, after further optimization, showed a consistent inhibition of both enzymatic activities in presence of diffent substituents [24]. Therefore, in the current study, we synthetised a new derivative (Z)-4-(2-(2-(2-oxoindolin-3-ylidene)hydrazinyl)thiazol-4-yl)benzonitrile (RMNC6) (Fig 1) that inhibits both RNase H and RDDP RT activities in the low micromolar range and used it to characterize the mechanism of action of this new class of dual inhibitors, combining biochemical and molecular modeling studies, supporting the hypothesis that isatin derivatives may access two different RT binding pockets involving highly conserved residues.

## Material and Methods

### Synthesis and characterization of RMNC6

Compound RMNC6 was synthesized and characterized as reported in S1 Appendix. Compound was solved in DMSO at 10 mM concentration and further diluted in water.

### Expression and purification of recombinant HIV-1 RT

#### HIV-1 RT group M subtype B

Heterodimeric RT was expressed essentially as described [25]. Briefly, *E*. *coli* strain M15 containing the p6HRT-prot vector was grown to an optical density at 600 nm of 0.7 and induced with 1.7 mM isopropyl β-D-1-thiogalactopyranoside (IPTG) for 4 h. Protein purification was carried out with a BioLogic LP system (Biorad), using a combination of immobilized metal affinity and ion exchange chromatography. Cell pellets were resuspended in lysis buffer (50 mM sodiumphosphate buffer pH 7.8, containing 0.5 mg/mL lysozyme), incubated on ice for 20 min, and after adding NaCl to a final concentration of 0.3 M, were sonicated and centrifuged at 30,000×g for 1 h. The supernatant was loaded onto a Ni^2+^-NTA-Sepharose column pre-equilibrated with loading buffer (50 mM sodium phosphate buffer pH 7.8, containing 0.3 M NaCl, 10% glycerol, and 10 mM imidazole) and washed thoroughly with wash buffer (50 mM sodium phosphate buffer pH 6.0, containing 0.3 M NaCl, 10% glycerol, and 80 mM imidazole). RT was eluted with an imidazole gradient in wash buffer (0–0.5 M). Fractions were collected, protein purity was checked by SDS-PAGE and found to be higher than 90%. The 1:1 ration between the p66/p51 subunits was also verified. Enzyme-containing fractions were pooled and diluted 1:1 with 50 mM sodium phosphate buffer pH 7.0, containing 10% glycerol; and then loaded into a Hi-trap heparin HP GE (Healthcare Lifescience) pre-equilibrated with 10 column volumes of loading buffer (50 mM sodium phosphate buffer pH 7.0, containing 10% glycerol and 150 mM NaCl). The column was then washed with loading buffer and the RT was eluted with Elute Buffer 2 (50 mM Sodium Phosphate pH 7.0, 10% glycerol, 1 M NaCl). Fractions were collected, protein was dialyzed and stored in buffer containing 50 mM Tris HCl pH 7.0, 25 mM NaCl, 1 mM EDTA, and 50% glycerol. Catalytic activities and protein concentrations were determined. Enzyme-containing fractions were pooled and aliquots were stored at −80°C.

#### HIV-1 RT group O

Recombinant HIV-1 group O RT was expressed and purified as previously described [26, 27]. It was obtained as a heterodimer composed of subunits of 66 and 51 kDa, with a polyhistidine tag at the C-terminus of p66. For this purpose, the RT p66 subunit encoded within a p66RTB expression vector was co-expressed with the HIV-1 protease in E. coli XL-1 Blue, and the obtained heterodimers were then purified by ionic exchange followed by chromatography on Ni2+-nitriloacetic acid agarose. The enzyme was quantified by active site titration before biochemical studies.

### Site-directed mutagenesis

Amino acid substitutions were introduced into the p66 HIV-1 RT subunit coded in a p6HRT-prot plasmid using the QuikChange mutagenesis kit by following the manufacturer’s instructions (Agilent Technologies Inc., Santa Clara, CA).

### HIV-1 DNA polymerase-independent RNase H activity determination

HIV RT-associated RNase H activity was measured as described [28] in 100 μL reaction volume containing 50 mM Tris-HCl buffer pH 7.8, 6 mM MgCl_2_, 1 mM dithiothreitol (DTT), 80 mM KCl, 0.25 μM hybrid RNA/DNA 5'-GAUCUGAGCCUGGGAGCU-Fluorescin-3' (HPLC, dry, QC: Mass Check) (available from Metabion) 5'-Dabcyl-AGCTCCCAGGCTCAGATC-3'(HPLC, dry, QC: Mass Check), increasing concentrations of inhibitors, whose dilution were made in water, and different amount of enzymes according to a linear range of dose-response curve: 20 ng of WT RT; 60 ng K103N RT; 37.5 ng V106A RT; 75 ng V108A RT; 5 ng Y181C RT; 50 ng Y188A RT; 30 ng Y188L RT; 100 ng E224A RT; 37.5 ng P225A RT; 20 ng P226A RT; 18 ng F227A RT; 30 ng L228A RT; 30 ng W229A RT; 10 ng M230A RT; 30 ng G231A RT; 62.5 ng N474A RT; 1 μg Y501A RT; 100 ng A502F RT; 37.5 ng A508V RT; 38 ng HIV-1 group O RT. The reaction mixture was incubated for 1 h at 37°C, stopped by addition of EDTA and products were measured with a multilabel counter plate reader Victor 3 (Perkin Elmer model 1420–051) equipped with filters for 490/528 nm (excitation/emission wavelength)

### HIV-1 RNA-dependent DNA polymerase activity determination

RNA-dependent DNA polymerase (RDDP) activity was measured as described [29] in 25 μL volume containing 60 mM Tris-HCl buffer pH 8.1, 8 mM MgCl_2_, 60 mM KCl, 13 mM DTT, 2.5 μM poly(A)-oligo(dT), 100 μM dTTP, increasing concentrations of inhibitors, whose dilution were made in water, and different amounts of enzymes according to a linear range of dose-response curve: 6 ng wt RT; 30 ng K103N RT; 12 ng V106ART; 19 ng V108A RT; 1.51.5 ng Y181C RT; 45 ng Y188A RT; 15 ng Y188L RT; 30 ng E224A RT; 15 ng P225A RT; 18 ng P226A RT; 23 ng F227A RT; 15 ng L228A RT; 30 ng W229A RT; 30 ng M230A RT; 15 ng G231A RT; 15 ng N474A RT; 15 ng Y501A RT; 15 ng A502F RT; 19 ng A508V RT; 38 ng HIV-1 group O RT. After enzyme addition, the reaction mixture was incubated for 30 min at 37°C and the stopped by addition of EDTA. Reaction products were detected by picogreen addition and measured with a multilabel counter plate reader Victor 3 (Perkin Elmer model 1420–051) equipped with filters for 502/523 nm (excitation/emission wavelength). The Yonetani-Theorell analysis was performed as described previously [30, 31].

### Determination of kinetic parameters of RNase H cleavage

Kinetic analysis of the DNA-polymerase independent RNase H activity was performed as already reported [32]. Briefly, in 100 μL reaction volume containing 50 mM Tris-HCl buffer pH 7.8, 6 mM MgCl_2_, 1 mM DTT, 80 mM KCl, fix amount of enzymes according to a linear range of dose-response curve: 20 ng of WT RT; 37.5 ng V106A RT; 75 ng V108A RT; 100 ng E224A RT; 100 RT ng A502F RT; 37.5 ng A508V RT, and increasing concentrations (from 0 to 500 nM) of hybrid RNA/DNA 5'-GAUCUGAGCCUGGGAGCU-Fluorescin-3'/ 5'-Dabcyl-AGCTCCCAGGCTCAGATC-3'. The reaction mixture was incubated for 1 h at 37°C, stopped by addition of EDTA and products were measured with a multilabel counter plate reader Victor 3 (Perkin Elmer model 1420–051) equipped with filters for 490/528 nm (excitation/emission wavelength). Results were represented according to Lineaweaver—Burke plot with the Sigmaplot10 software. Velocity (ν) was expressed as fmoles/min.

### Detection of protein inhibitor interactions by differential scanning fluorimetry

Thermal stability assays were performed according to Nettleship et al. [33]. In a LightCycler 480 96-well plate (Roche) we incubated 10 μM inhibitor, in 50 μl of reaction buffer containing 20 mM HEPES, pH 7.5, 10 mM MgCl_2_, 100 mM NaCl, 300 nM of HIV-1 RT and a 1:1000 dilution of Sypro Orange dye (Invitrogen). The mixture was heated from 30 to 90°C in increments of 0.2°C. Fluorescence intensity was measured using excitation and emission wavelengths of 483 and 568 nm, respectively. Changes in protein thermal stability (ΔTm) upon inhibitor binding were analyzed by using the LightCycler 480 software. All assays were performed in triplicate.

### Molecular modeling

#### Ligand preparation

Theoretical 3D model of RMNC6 was built with the Maestro software (Schrödinger LLC., Maestro GUI, New York, NY, USA, 2012). The inhibitor structure was optimized by energy minimization carried out using the MMFFs force field [34], the GB/SA [35] water implicit solvation model and the Polak-Ribier Coniugate Gradient (PRCG) method, converging on gradient with a threshold of 0.05 kJ (mol*Å)^-1^.

#### Protein preparation

The coordinates for RTs were taken from the RCSB Protein Data Bank [36] (PDB codes 1vrt [37], 2zd1 [38], 1ep4 [39], 3qo9 [40], 1rti [37], 1tv6 [41], 3lp2 [42]. The proteins were prepared by using the Maestro Protein Preparation Wizard. Original water molecules were removed and termini were capped. The bond orders and formal charges were added for hetero groups, and all the hydrogen atoms were added in the structure. Missing atoms and residues were included. After preparation, the structures were refined in order to optimize the hydrogen bond network using OPLS_2005 [43] force field. The minimization was terminated when the energy converged or the RMSD reached a maximum cut-off of 0.30 Å.

#### Docking protocol

Molecular docking studies were performed using QMPL workflow protocol [44] (Schroedinger Suite 2014, Schrodinger Inc, Portland, USA). Grids were defined around the refined structure by centering on the residue W229 (located in the NNRTI binding pocket) and Q500 (located in the RNase H domain) and fixing the box volume at 97,336 Å^3^, covering the whole p66 and most of p51 subunit. The extra precision (XP) docking algorithm was applied for scoring theoretical poses. The other settings were left as default. The same protocol was applied for all the simulations.

#### Post docking protocol

Ten thousand steps of the Polak-Ribier conjugate gradient (PRCG) minimization method were conducted on the top ranked theoretical complexes using OPLS_2005 force field. The optimization process was performed up to the derivative convergence criterion equal to 0.01 kJ/(mol*Å)^1^. The binding free energies (ΔG(Bind)) were computed applying molecular mechanics and continuum solvation models with the molecular mechanics generalized Born/surface area (MM-GBSA) method [45].

## Results and Discussion

### Characterization of RMNC6 as a dual inhibitor acting on the RDDP and RNase H activities of HIV-1 RT

We synthetized a new derivative (Z)-4-(2-(2-(2-oxoindolin-3-ylidene)hydrazinyl)thiazol-4-yl)benzonitrile (RMNC6) (Fig 1) that, when tested on RT from the HIV-1 group M inhibited both RNase H and RDDP activities with IC_50_ values of 1.4 and 9.8 μM, respectively.

**Fig 2:**
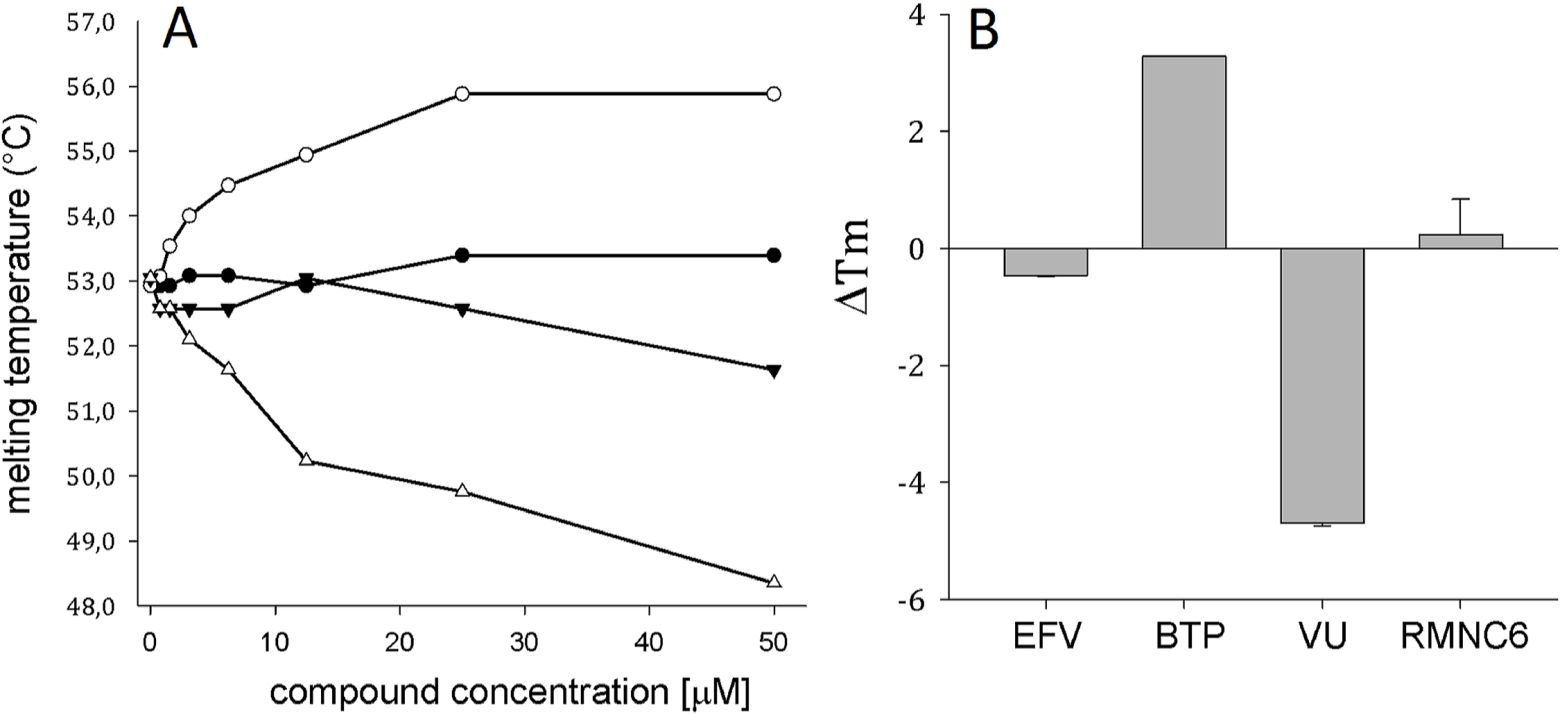
Effect of RT inhibitors on the thermal stability of p66/p51 HIV-1 RT. (A). The melting temperature of HIV-1 RT was measured by differential scanning fluorimetry in the presence of increasing concentrations of different inhibitors: (▼) Efavirenz (EFV), (○) β-thujaplicinol (BTP), (Δ) 2-(3, 4-dihydroxyphenyl)-5, 6-dimethylthieno[2, 3-d]pyrimidin-4(3H)-one (VU) and (●) RMNC6. (B). Maximum HIV-1 RT thermal shift (ΔTm) observed in the presence of 50 μM concentration of compounds. ΔTm values are the average of triplicate analysis, standard deviations are indicated as bars.

Since the mode of action of previously identified dual RT inhibitors was not determined, we decided to investigate the mechanism of RT inhibition by RMNC6 in comparison with available RNase H inhibitors and NNRTIs. Since many RNase H inhibitors act as chelators of cations needed in the active site (e.g. diketo acid derivatives [46]), we firstly analyzed chelating potential of RMNC6 by measuring its UV spectra in the absence and in the presence of magnesium. Results showed that RMNC6 maximum of absorbance did not shift in the presence of 6 mM MgCl_2_, excluding the involvement of chelation in the mechanism of action (data not shown). RNase H inhibitors such as vynologous ureas (VUs) are known to destabilize the RT heterodimer by binding to an allosteric pocket in the RNase H domain at the interface between p66 and p51 subunits [47]. Therefore, we examined alterations in HIV-1 RT thermal stability by differential scanning fluorimetry [48] in the presence of increasing concentrations of RMNC6 as well as known inhibitors such as the NNRTI Efavirenz (EFV), the RNase H active-site inhibitor hydroxytropolone β-thujaplicinol (BTP) and the allosteric RNase H inhibitor 2-(3, 4-dihydroxyphenyl)-5, 6-dimethylthieno[2, 3-d]pyrimidin-4(3H)-one (VU) [49]. In agreement with previous studies [47, 50], the RNase H active-site inhibitor BTP, that has been shown to stabilize the RT against thermal denaturation [50], caused a Tm increase of < 2.0°C in the presence of Mg^2+^, while the interface inhibitor VU, that has been shown to destabilize the HIV-1 RT [47] decreased the Tm by 0.5−5.5°C (Fig 2). In contrast, RMNC6 did not affect significantly RT thermal stability, showing a similar behavior to EFV and suggesting that RMNC6 may have an allosteric binding mode different from VU but possibly similar to the one shown by EFV (Fig 2).

### Effects of RMNC6 on efavirenz inhibition and susceptibility of NNRTI-resistant RTs

**Fig 3:**
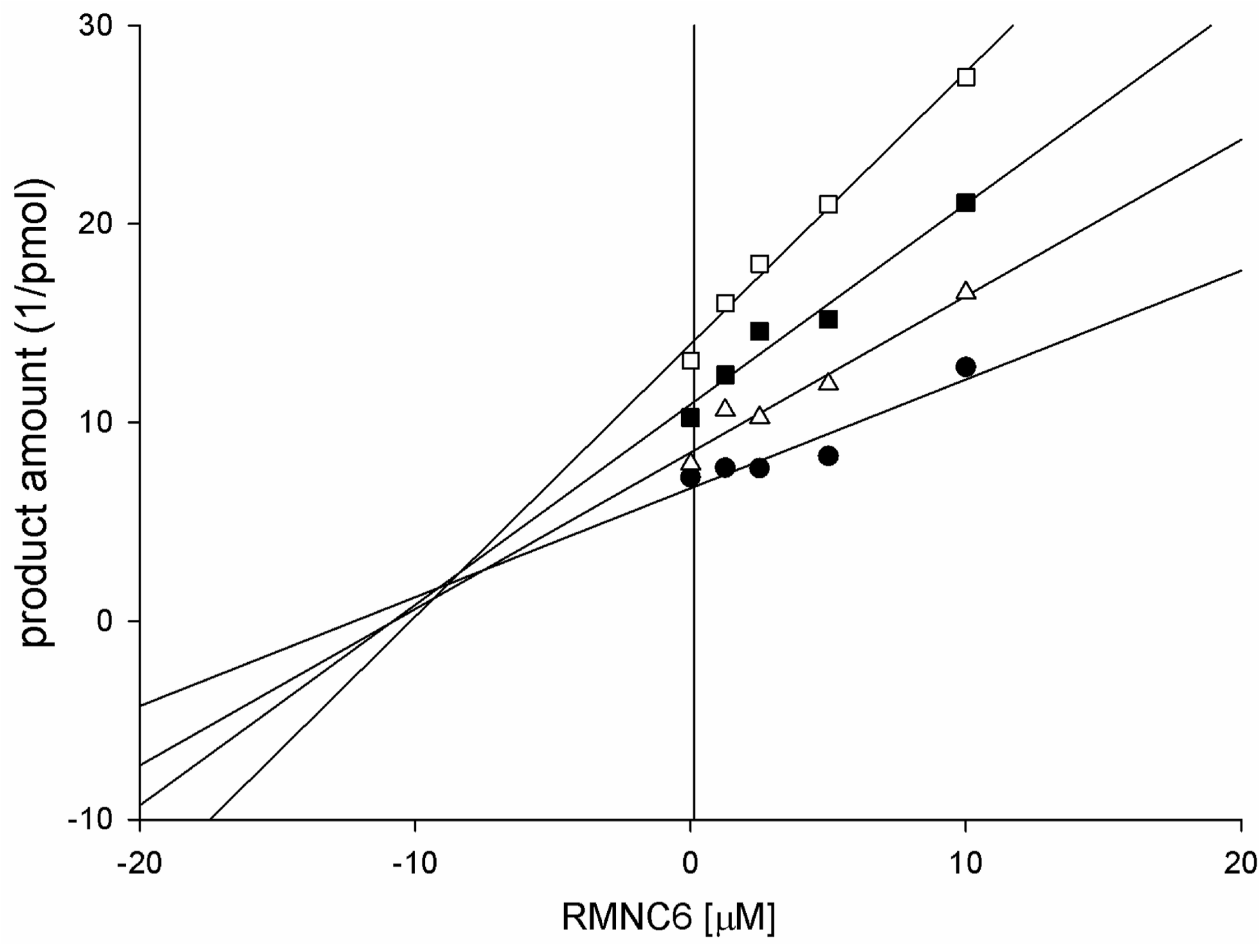
Yonetani-Theorell plot of the combination of RMNC6 and EFV on the HIV-1 RT RNA- dependent DNA polymerase activity. HIV-1 RT was incubated in the presence of RMNC6 alone (●) or in presence of different concentrations of EFV: 4 nM (Δ), 8 nM (■) and 16 nM (☐).

To investigate the RMNC6 mode of action with respect to EFV, we performed a Yonetani-Theorell analysis [30] on the combined effects of RMNC6 and EFV on RDDP function. Such an analysis reveals whether simultaneous binding (or inhibition) of two compounds is possible or not. Results (Fig 3) showed that RMNC6 and EFV inhibition are not mutually exclusive. However, it is worth to note that the calculated interaction constant α had the value of 1.2, suggesting a negative interference between the two compounds (i.e. EFV binding has a negative influence on RMNC6 binding, and vice versa). Furthermore, we tested RMNC6 against several mutants conferring resistance to NNRTIs such as K103N, Y181C and Y188L, and against the HIV-1 group O RT which shows natural resistance to NNRTIs [51] due to the presence of the amino acid substitutions A98G, V179E and Y181C (S2 Appendix). The DNA polymerase activity of HIV-1 group O RT as well as the one of wild-type and mutant of HIV-1 group M subtype B RTs were all susceptible to RMNC6 in RDDP assays (IC_50_ values in the range 5.4–20.1 μM). In contrast, HIV-1 group O RT and mutants K103N and Y188L showed decreased susceptibility to EFV (7.7- to 9.2-fold increases in the IC_50_ values relative to the wild type HIV-1 group M subtype B RT) (Table 1). These observations suggest different modes of action for RMNC6 and EFV.

**Table 1:** Susceptibility to RMNC6 of wt and NNRTI-resistant HIV-1 RTs measured in RNase H and RDDP activity assays.

	RMNC6		BTP	EFV
RT	RNase H	RDDP	RNase H	RDDP
	IC_50_ (μM)^a^	IC_50_ (μM)^b^	IC_50_ (μM)^a^	IC_50_ (nM)^b^
**Wt**	1.3 ± 0.3	9.8 ± 1.4	0.19 ± 0.03	23 ± 2.7
**K103N**	2.3 ± 0.1	13.6 ± 1.0	0.22 ± 0.08	176 ± 25
**Y181C**	2.1 ± 0.6	5.4 ± 0.3	0.23 ± 0.05	49.7 ± 9.1
**Y188L**	1.6 ± 0.5	16.5 ± 3.7	0.08 ± 0.05	198 ± 60
**Group O**	8.5 ± 2.6	20.1 ± 7.4	0.91 ± 0.01	212 ± 46

^a^Concentration required to inhibit HIV-1 RT-associated RNase H activity by 50%. Data were obtained by three independent experiments (reported as average ± standard deviation).

^b^Concentration required to inhibit HIV-1 RT-associated RDDP activity by 50%. Data were obtained by three independent experiments (reported as average ± standard deviation).

Interestingly, the HIV-1 group O RT was also inhibited by BTP and RMNC6 in RNase H activity assays, although IC_50_ values were 5 to 6.5 times higher than those obtained with the prototypic wild-type HIV-1 group M subtype B RT. These effects could be attributed to differences that affect the overall structure of the RNase H domain in both enzymes. Previous studies showed that HIV-2 RT was about 3.7 times less susceptible to BTP than the HIV-1 enzyme [52], while the crystal structure of BTP bound to HIV-1 group M subtype B RT revealed that major interactions involved in inhibitor binding affected RNase H active site residues [42]. However, RNase H active site residues are conserved in all HIV-1 and HIV-2 clades. Outside the active site, the most significant differences between group M subtype B and group O HIV-1 RTs are found around positions 460–471, 482–492, and 502–508 (S2 Appendix).

### Blind docking analysis

To achieve further insights into the RMNC6 binding mode, we performed blind docking studies on the wt HIV-1 group M subtype B RT heterodimer and RMNC6, by using the QM-polarized ligand docking protocol [44] and using Glide version 6.2, Qsiteversion6.2, Jaguar 8.3 and Maestro 9.7 (Schroedinger Suite 2014, Schrodinger Inc, Portland, USA).

**Fig 4:**
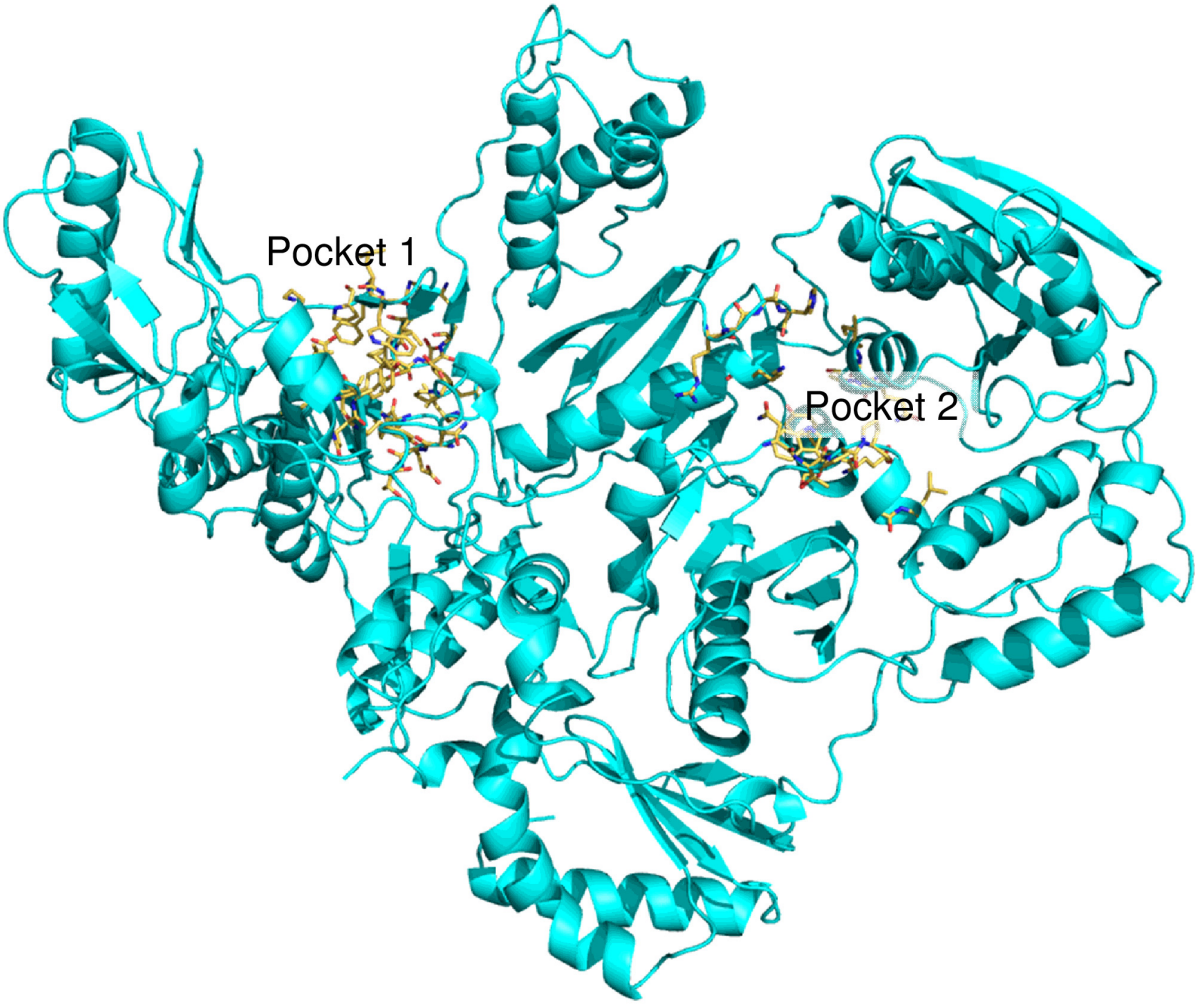
Binding sites of RMNC6 individuated after blind docking experiments on the whole wt HIV-1 RT structure.

Due to the flexibility of the target and different shapes of known inhibitors [18], we decided to carry out ensemble docking experiments. The major conformational changes in the NNRTI binding pocket were taken into account to cluster the available RT complexes. In particular, the orientation of amino acid residues Y181, Y188, Y183 and primer grip β12-β13 hairpin were considered [53]. A representative of each different cluster was picked and the three-dimensional structure of HIV RT was retrieved from the Protein Data Bank. The obtained [RMNC6•RT] complexes were then subjected to a post-docking procedure, based on energy minimization, and successive binding free energies calculation. The binding free energies (ΔG(Bind)) were obtained by applying molecular mechanics and continuum solvation models using the molecular mechanics generalized Born/surface area (MM-GBSA) method [45]. By comparing the ΔG-MMGBSA values, we could assert that blind docking calculations indicated the presence of two energetically favored binding pocket sites for isatin derivative (Table 2) (Fig 4).

**Table 2:** Ensemble docking results: binding free energies of [RMNC•RT] complexes. The most likely binding poses are indicated in bold.

RT structure (pdb code)	Pocket	ΔG_MMGBSA (Kcal/mol)
1ep4	1	-41.23
1ep4	2	-36.22
3lp2	1	-44.85
3lp2	2	-42.58
1rti	1	-33.26
1rti	2	-40.49
1tv6	1	**-51**
1tv6	2	-**48.74**
1vrt	1	-35.9
1vrt	2	-32.44
2zd1	1	-41.96
2zd1	2	-32.06
3q09	1	-39.25
3q09	2	-38.48

**Fig 5:**
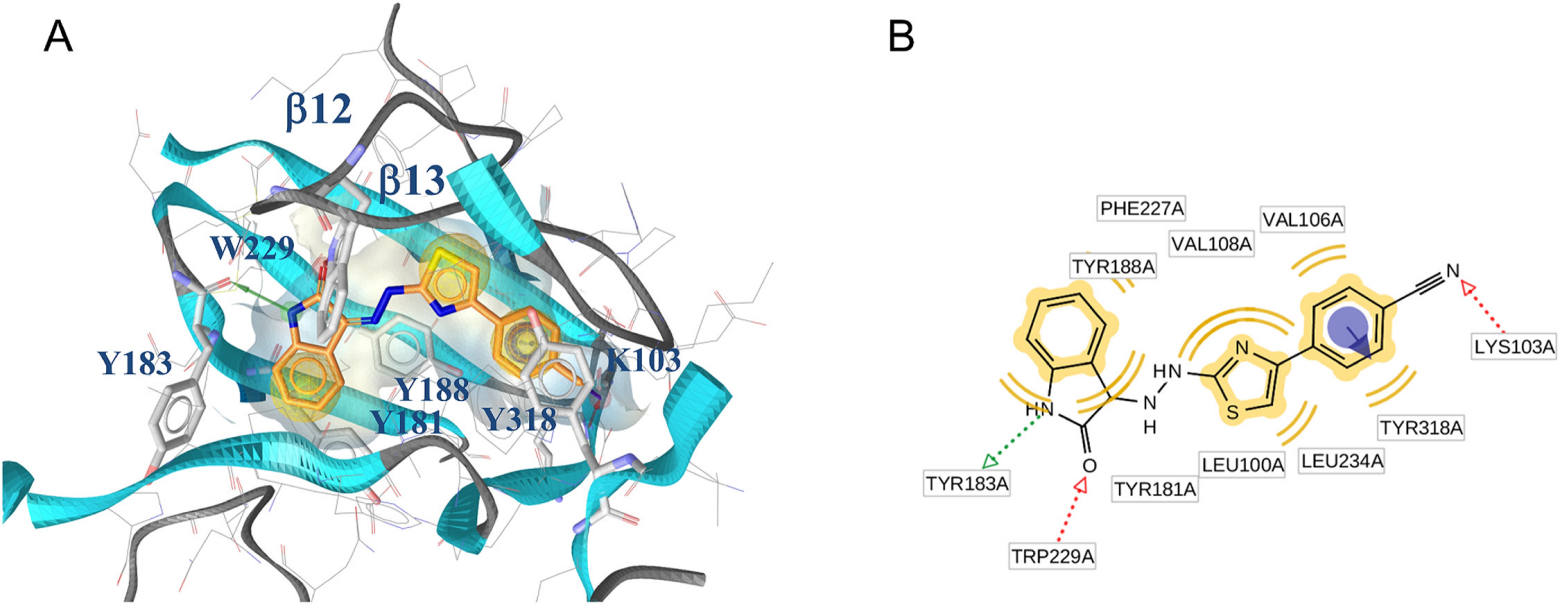
Putative binding mode of RMC6 and critical residues individuated for RMNC6 binding in the pocket 1. (A) binding mode; (B) 2D depiction of RMNC6 and its respective interactions with RT residues: pale yellow sphere indicates hydrophobic interactions with lipophilic residues. Red arrow indicates an hydrogen bond (HB) acceptor interaction, green HB donor, while the violet sphere represents the aromatic π-π stacking interaction.

The first and most energetically favored binding site (pocket 1) (Table 2) is located close to the DNA polymerase catalytic center and is contiguous to the NNRTI binding pocket, having an “L shape”. The most stable pose (Fig 5) involves amino acids L100, K103, V106, V108, Y183, Y188, L234, W229 and Y318. Furthermore, according to the model, different residues of the hairpin constituted by the β12 and β13 sheet [54] could be involved with RMNC6 binding in the hypothesized orientation.

**Fig 6:**
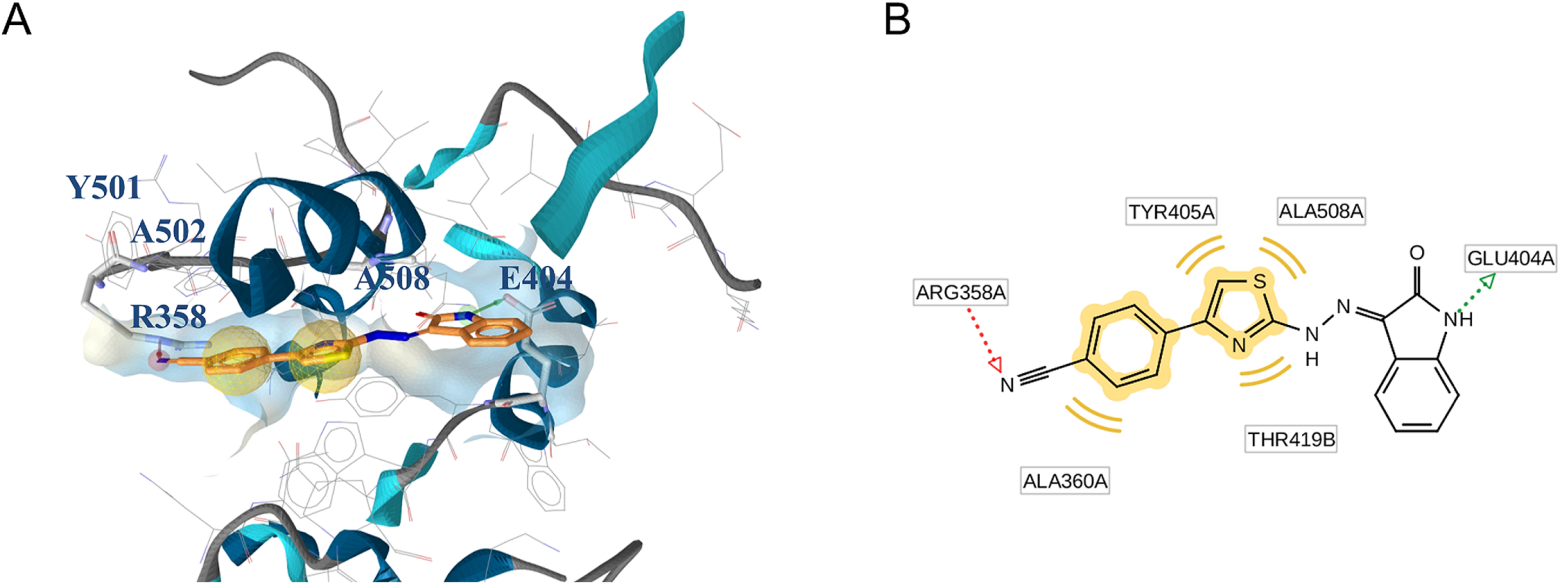
(A) Putative binding mode of RMNC6 and critical residues individuated for RMNC6 binding in the pocket 2. (B) 2D depiction of RMNC6 and its respective interactions with RT residues.

The second putative binding pocket (pocket 2) is located in the RNase H domain, between the RNase H active site and the primer grip region, close to the p66/p51 interface. Docking modeling suggests that, in this site, RMNC6 could be partially sandwiched between different secondary structural units, such as namely β21 strand and the αH helix in p51, and the αB helix in p66 (residues 500–508) (Fig 6).

Overall, such *in silico* analysis supports alternative modes of action for RMNC6, since the drug could bind (i) preferentially only to one of the two pockets and have both short- and long-range effects; or (ii) to both sites and act by short-range effects on the two functions. According to the first mode of action, RMNC6 would inhibit RDDP and RNase H activities through binding into the sole pocket 1, by having a short-range inhibitory effect on RDDP activity and a long-range inhibitory effect on RNase H activity. Hence, it would act differently from classical NNRTIs such as nevirapine and EFV that were shown to destabilize the 3’-end of the DNA primer in the DNA polymerase active site and promote RT-mediated polymerase-independent RNase H cleavages [11]. Alternatively, according to the first mode of action, RMNC6 could bind to the sole pocket 2 and it would have a short-range inhibitory effect on RNase H activity and a long-range effect on the RDDP activity. Differently, according to the second mode of action RMNC6 would bind to both individual pockets and its activity would be due to short-range inhibition effects, so that its binding to each site would be responsible for the inhibition of one function. To dissect this diverse scenario of possibilities we performed kinetic and site-directed mutagenesis studies.

### Influence of the amino acid residues in pocket 1 on HIV-1 RT inhibition by RMNC6

Considering the size of pocket 1, its surface was explored in detail by site-directed mutagenesis. Since the hairpin comprising strands β12 and β13 was identified as potentially involved in RMNC6 binding alanine-scanning mutagenesis was performed for amino acids 224–231. Mutants V108A and V106A were also obtained since Val108 and Val106 were identified as potentially important for inhibitor binding. The susceptibility of mutant RTs to RMNC6 was also tested in RNase H and RDDP activity assays, using BTP and EFV as positive controls (Table 3). Interestingly, the E224A RT showed a 6-fold reduced susceptibility to RMNC6 in RNase H assays, while mutant enzymes such as V108A and V106A showed IC_50_ values 4 and 10 times higher, respectively, in comparison with the wt RT. In contrast, we did not observed major differences in the IC_50_ values obtained with other mutant RTs (Table 3).

**Table 3:** Effects of selected amino acid substitutions in pocket 1 of HIV-1 RT in the susceptibility to RMNC6 in RNase H and RDDP activity assays.

	RMNC6		BTP	EFV
RT	RNase H	RDDP	RNase H	RDDP
	IC_50_ (μM)^a^	IC_50_ (μM)^b^	IC_50_ (μM)^a^	IC_50_ (nM)^b^
**V106A**	5.3 ± 0.6 (4.1)^c^	25.1 ± 2.3 (2.5)	0.16 ± 0.03 (0.8)	35.4 ± 2.2 (1.5)
**V108A**	13.1 ± 2.5 (10)	33.0 ± 5.2 (3.4)	0.18 ± 0.04 (0.9)	21.3 ± 3.6 (0.9)
**Y188A**	3.3 ± 1.5 (2.5)	20.9 ± 4.0 (2.1)	0.19 ± 0.08 (1.0)	28.3 ± 7.4 (1.2)
**E224A**	7.9 ± 1.1 (6.1)	13.8 ± 4.2 (1.4)	0.15 ± 0.03 (0.8)	24.9 ± 0.7 (1.1)
**P225A**	1.7 ± 0.2 (1.3)	3.1 ± 0.3 (0.3)	0.13± 0.06 (0.7)	19.5 ± 2.9 (0.8)
**P226A**	1.9 ± 0.1 (1.5)	18.9± 0.7 (1.9)	0.29 ± 0.08 (1.5)	18.9 ± 2.4 (0.9)
**F227A**	0.7 ± 0.1 (0.5)	15.2 ± 0.9 (1.5)	0.22 ± 0.04 (1.2)	61.5 ± 1.4 (2.7)
**L228A**	1.5 ± 0.1 (1.1)	13.1 ±1.9 (1.3)	0.10 ± 0.06 (0.5)	21.8 ± 3.8 (1.0)
**W229A**	1.9 ± 0.5 (1.5)	15.4 ± 2.8 (1.6)	0.15 ± 0.08 (0.8)	22.9 ± 5.2 (1.1)
**M230A**	0.5 ± 0.1 (0.4)	15.0 ± 3.5 (1.5)	0.14 ± 0.04 (0.7)	29.1 ± 2.2 (1.3)
**G231A**	1.7 ± 0.4 (1.3)	10.8 ± 2.4 (1.1)	0.25 ± 0.03 (1.3)	67.7 ±3.5 (2.9)

^a^Concentration required to inhibit HIV-1 RT-associated RNase H activity by 50% obtained by three independent experiments (reported as average ± standard deviation).

^b^Concentration required to inhibit HIV-1 RT-associated RDDP activity by 50% obtained by three independent experiments (reported as average ± standard deviation).

^c^Fold of increase with respect to wt RT.

When the RDDP function of mutant RTs was assayed, a similar pattern emerged, even though the extent of the reduction in the IC_50_ values was lower (1.4, 3.3 and 2.5 μM for E224A, V106A and V108A RTs, respectively). Hence, the results of the assays with the mutant RTs did not provide incontrovertible evidence of RMNC6 binding. However, the results could not exclude the RMNC6 binding to pocket 1. In fact, given the amplitude of the pocket and the high RT flexibility, the very limited IC_50_ reduction towards RDDP function observed could be determined by a different orientation of RMNC6 while accommodating in pocket 1, as a response to the introduced amino acid changes. It is worth noting that the kinetic analysis of the interaction between RMNC6 and EFV, showed a negative interference between the two compounds, even if they are not kinetically mutually exclusive, supporting the possibility that RMNC6 could accommodate in the large pocket 1 even in the presence of EFV.

### Influence of the amino acid residues in pocket 2 on HIV-1 RT inhibition by RMNC6

Pocket 2, located in the RNase H domain, was already predicted [20] and investigated in the context of hydrazone derivatives binding studies [21, 22]. According to our model, when bound to this site, RMNC6 might nudge the RNase H domain to a position in which the active site might no longer be able to catalyze hydrolysis cleavage of the RNA strand in the of RNA:DNA duplex. To investigate possible RMNC6 binding into this pocket, we mutated residues Ala502 to Phe and Ala508 to Val in an attempt to reduce the space available for RMNC6 accommodation. In addition, we also mutated residues Asn474 and Tyr501 to Ala, since they were previously shown to be involved in the interactions between diketo acids and the RNase H domain [32] and, even if not predicted to be directly having contacts with this isatin inhibitor, these two residues are known to play an important structural role in the functional geometry of the region, as part of the RNase H primer grip motif. Mutated residues are also highly conserved, as indicated by the drastic reduction in viral infectivity of mutant viruses [55]. Hence, we tested RMNC6 effects on both RNase H and RDDP functions of mutant RTs, using BTP and EFV as positive controls (Table 4). The RNase H function of mutant RTs A502F and A508V showed 4- to 10-fold reduced susceptibility to RMNC6 in RNase H assays, while individual mutations of N474A and Y501A caused full resistance to the isatin derivative. Interestingly, BTP showed a different profile and was an efficient inhibitor of all mutants except N474A that had an IC_50_ value 4.5 times higher than the one obtained with the wt RT.

**Table 4:** Effects of selected amino acid substitutions in pocket 2 of HIV-1 RT in the susceptibility to RMNC6 in RNase H and RDDP activity assays.

	RMNC6		BTP	EFV
RT	RNase H	RDDP	RNase H	RDDP
	IC_50_ (μM)^a^	IC_50_ (μM)^b^	IC_50_ (μM)^a^	IC_50_ (nM)^b^
**N474A**	> 100 (> 77)^c^	12.7 ± 1.7 (1.3)	0.92 ± 0.01 (4.8)	20.1 ± 5.1 (1.4)
**Y501A**	> 100 (> 77)	6.6 ± 0.1 (0.7)	0.07 ± 0.002 (0.4)	24.1 ± 3.5 (1.7)
**A502F**	13.0 ± 0.7 (10.0)	17.1± 2.1 (1.8)	0.17 ± 0.03 (0.9)	22.3 ± 1.5 (1.6)
**A508V**	6.5 ± 0.7 (5.0)	19.3 ± 2.8 (2.0)	0.16 ± 0.05 (0.8)	24.7 ± 2.4 (1.8)

^a^Concentration required to inhibit HIV-1 RT-associated RNase H activity by 50% obtained by three independent experiments (reported as average ± standard deviation).

^b^Concentration required to inhibit HIV-1 RT-associated RDDP activity by 50% obtained by three independent experiments (reported as average ± standard deviation).

^c^Fold of increase with respect to wt RT.

Noteworthy, these amino acidic substitutions did not affect RMNC6 susceptibility in RDDP assays. These results strongly support the hypothesis that RMNC6 interacts with pocket 2 and that this binding is responsible for short-range inhibition of the RNase H function. The data are also consistent with the possibility of RMNC6 binding to both pockets 1 and 2 and acting by two different short-range effects. In fact, in these mutant RTs, RMNC6 binding to pocket 1 could be responsible for the inhibition of the RDDP activity, while RMNC6 binding to pocket 2 could be impeded. The amino acid sequence alignment of HIV-1 RTs of group O and group M subtype B [55, 56] reveals differences at residues forming pocket 2. Thus, at position 404, 502, 508 and 511, the HIV-1 group O RT contains Asp, Val, Ser and Thr, respectively, while the prototypic group M subtype B enzyme has Glu404, Ala502, Ala508 and Asp511. The 6.5 folds reduction in RMNC6 susceptibility obtained with HIV-1 group O RT in RNase H activity assays (Table 1) is consistent with the effects observed with single-mutants A502F or A508V (Table 4), since amino acid substitutions found in the HIV-1 group O RT would also contribute to reducing the size of the putative RMNC6 binding pocket in the RNase H domain. In addition, the NNRTI binding pocket of the HIV-1 group M subtype B RT differs from that of the group O enzyme mainly at the 181 position (Cys in HIV-1 group O RT and Tyr in HIV-1 group M subtype B RT), but this does not affect the inhibitory efficiency of RMNC6. Taken together, our data reveal a relevant role for pocket 2 in RMNC6 binding.

### RNase H cleavage kinetics

With respect to RMNC6 binding to pocket 1, the observed partial loss in potency of RNase H inhibition observed for V106A, V108A and E224A RTs (Table 3) supported two different hypotheses. According to the first one, reduced potency could be due to an impairment of a long-range effect of RMNC6 bound into pocket 1. According to the second one, the single mutations can lead to a long-range modification of the pocket 2 or the template-primer accommodation that alters the compounds binding into pocket 2. To examine the latter hypothesis, we asked if these mutations could influence the RNase H function and hence evaluated the RNase H catalytic efficiency of all DNA polymerase domain mutants that exhibited a reduced RNase H inhibition by RMNC6. It is worth to note in this context that residue Glu224 is highly conserved since it is involved in the DNA polymerase primer grip motif [6]. In addition, among the mutated residues in the RNase H domain, Asn474 and Tyr501 are also highly conserved, since they are also part of the RNase H primer grip, and whose mutation has non conservative substitutions at those positions have been shown to decrease the RT RNase H activity [37, 41]. Therefore, we tested whether mutant A502F and A508V RTs had altered RNase H kinetics cleavage efficiency. Results showed a consistent reduction in the *k*_cat_ values with respect to wt RT for all mutant RTs, and a slight reduction in template-primer K_m_ values for A508V, V106A, V108A and E224A RTs, resulting in a reduced efficiency (k_cat_/K_m_) for V108A, E224A and A502F RTs (2-, 3 and 12-fold, respectively) (Table 5). Overall, these results support the hypothesis of long-range effects of mutations in the RT polymerase domain on the RNase H activity that can extend to RMNC6 binding pocket 2.

**Table 5:** Kinetic parameters of RNase H cleavage for wt and mutant HIV-1 RTs.

RT	^a^*k*_cat_ (min^-1^)	^a^*K*_m_ (nM)	*k*_cat_/*K*_m_
**Wt**	113 ± 1.4	127 ± 5.0	0.89
**V106A**	52.6 ± 5.6	69.0 ± 11.0	0.76
**V108A**	19.4 ± 3.3	48.8 ± 3.9	0.40
**E224A**	21.2±4.3	78.6 ± 14.0	0.27
**A502F**	20.8 ± 1.0	273 ± 18	0.076
**A508V**	51.7 ± 4.9	74.9 ± 6.8	0.68

^a^Data were obtained from three independent experiments (reported as average ± standard deviation)

## Conclusions

Our study demonstrates that RMNC6 is a novel dual function RT inhibitor that has an allosteric binding mode, different from that shown by known NNRTIs. Our results support the hypothesis that RMNC6 binds two different sites in HIV-1 RT. This double-site binding mode seems to confirm that it could be possible to target both RT-associated functions by a single molecule, retaining full potency of inhibition on drug-resistant mutant RTs such as K103N, Y181C and Y188L. RNase H inhibition by RMNC6 seems to be mainly due to its interaction with pocket 2, close to the RNase H active site. The long-range effects on RNase H inhibition produced by RMNC6, and observed when amino acids in the polymerase domain were mutated, are probably due to an alteration of the substrate binding pocket in the RNase H domain that, in turn, alters RMNC6 binding or efficacy. On the contrary, HIV-1 RT-associated RDDP inhibition by RMNC6 seems to be related to its binding in the polymerase domain. However, the size of the pocket and the plasticity of the enzyme may be responsible for the compound retained RDDP inhibition on mutant RTs. Overall, dual RT-associated functions inhibition by compounds binding to two sites is an appealing possibility to strongly reduce drug resistance occurrence. Furthermore, since pocket 2 is particular for HIV RT, compounds that target this site will not interfere with human RNase H1. These results prompt us to undergo further studies to better define the drug interactions within these two pockets in order to perform a rational drug design of dual inhibitors acting on both binding sites.

## Supporting Information

S1 Appendix Synthesis and characterization of RMNC6. (DOCX)

S2 Appendix Amino acid sequence alignment of HIV-1 group M subtype B and group O RTs. (DOCX)
