# Supplementary material for: Ribonuclease H/DNA Polymerase HIV-1 Reverse Transcriptase Dual Inhibitor: Mechanistic Studies on the Allosteric Mode of Action of Isatin-Based Compound RMNC6
Source: PLoS One. 2016 Jan 22;11(1):e0147225. doi: 10.1371/journal.pone.0147225 (PMC4723341; doi:10.1371/journal.pone.0147225)
Supplement: S1 Appendix — (DOCX) [file pone.0147225.s001.docx]

**Ribonuclease H/DNA polymerase HIV-1 reverse transcriptase dual inhibitor: mechanistic studies on the allosteric mode of action of isatin-based compound RMNC6**

Angela Corona^1^, Rita Meleddu^1^, Francesca Esposito^1^, Simona Distinto^1^, Giulia Bianco^1^, Takashi Masaoka^2^, Elias Maccioni^1^, Luis Menéndez-Arias^3^, Stefano Alcaro^4^, Stuart F.J. Le Grice^2^ and EnzoTramontano^1#^

**S1 Appendix. Synthesis and characterization of RMNC6**

2,3-dihydro-1H-indole-2,3-dione was reacted with thiosemicarbazide in 2-propanol to give 2-oxo-2,3-dihydro-1H-indol-3-thiosemicarbazone . Equimolar amounts of III and 4-(2-bromoacetyl)benzonitrile were then stirred in 2-propanol to give the desired final compounds (figure S1 A).

##

## figure S1 A. Synthesis of RMNC6. 2,3-dihydro-1H-indole-2,3-dione (I)was reacted with thiosemicarbazide (II) in 2-propanol to give 2-oxo-2,3-dihydro-1H-indol-3-thiosemicarbazone (III). Equimolar amounts of III and 4-(2-bromoacetyl)benzonitrile(IV) were then stirred in 2-propanol to give the desired final compounds.

The purified compound was then characterized by means of both analytical and spectroscopic methods.

In particular NMR spectra were measured in DMF-d7 solvent at 278.1 K temperature on a Bruker AVANCE III spectrometer (figure S1 B). In the signal assignments the proton and carbon chemical shifts are referred to the solvent (^1^H: δ = 8.03 ppm, ^13^C dowfield methyl signal: δ=34.89 ppm respectively). In the ^15^N chemical shift assignments we applied the spectrometer’s digital reference which is calibrated to liq. NH_3_δ= 0 ppm.

Z configuration was supported by NMR: This was based on the selective NOE experiment, where we observed NOE interaction between the indole NH and CH protons, while no correlation was seen between the indole CH and =N-NH- hydrogens.

C18H11N5OS yellow-orange solid, M.p>250°C, MS (m/z) 345, yield 99%; ^1^H NMR (DMF) δ(ppm): 13.54 (s; 1H); 11.42 (s; 1H); 8.23-8.17 (m; 2H); 8.04 (s; 1H); 7.97-7.93 (m; 2H); 7.62 (d; *J*: 7.6 Hz; 1H); 7.40 (tm; *J*: 7.6 Hz; 1H); 7.15 (tm; *J*: 7.6 Hz; 1H); 7.08 (d; *J*: 7.6 Hz; 1H)

^13^C NMR (DMF) δ(ppm): 167.2; 163.9; 150.1; 142.2; 138.8; 133.2; 133.0; 131.0; 126.8; 122.9; 120.4; 120.3; 119.3; 111.6; 110.8; 110.6

^15^N NMR (DMF) δ(ppm): 155.9; 135.6.

**Figure S1 B. Structure of RMNC6 compound with NMR signals labeled**. ^1^H NMR (DMF) δ(ppm) in red; ^13^C NMR (DMF) δ(ppm) in blue; ^15^N NMR (DMF) δ(ppm) in green.
