# Supplementary material for: Ribonuclease H/DNA Polymerase HIV-1 Reverse Transcriptase Dual Inhibitor: Mechanistic Studies on the Allosteric Mode of Action of Isatin-Based Compound RMNC6
Source: PLoS One. 2016 Jan 22;11(1):e0147225. doi: 10.1371/journal.pone.0147225 (PMC4723341; doi:10.1371/journal.pone.0147225)
Supplement: S2 Appendix — (DOCX) [file pone.0147225.s002.docx]

**Ribonuclease H/DNA polymerase HIV-1 reverse transcriptase dual inhibitor: mechanistic studies on the allosteric mode of action of isatin-based compound RMNC6**

Angela Corona^1^, Rita Meleddu^1^, Francesca Esposito^1^, Simona Distinto^1^, Giulia Bianco^1^, Takashi Masaoka^2^, Elias Maccioni^1^, Luis Menéndez-Arias^3^, Stefano Alcaro^4^, Stuart F.J. Le Grice^2^ and EnzoTramontano^1#^

**S2 Appendix. Amino acid sequence alignment of HIV-1 group M subtype B and group O RTs**


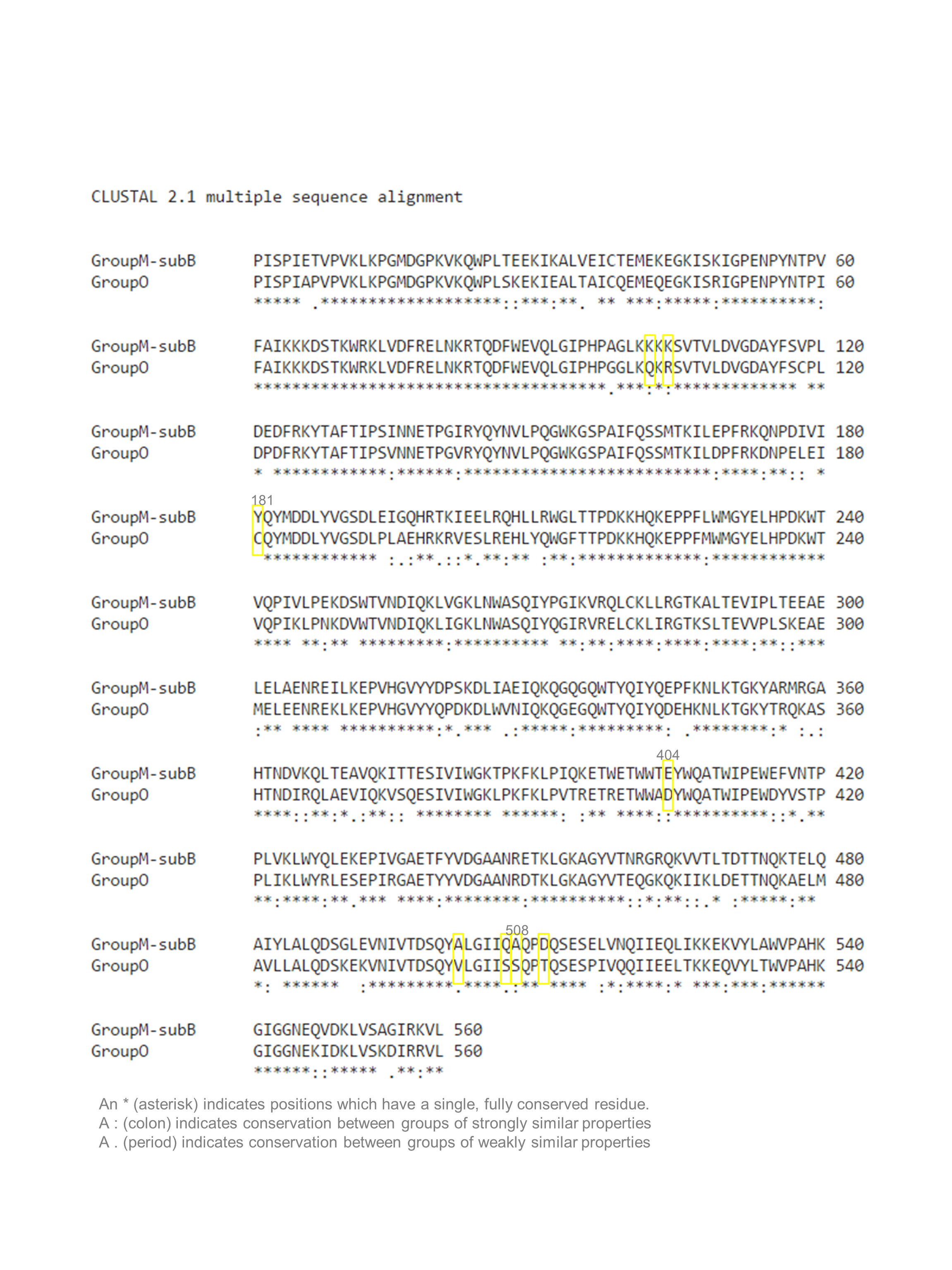


**Figure S2. Amino acid sequence alignment of HIV-1 group M subtype B and group O RTs**. Residues located in the RMNC6 putative binding sites whose role has been studied by site-directed mutagenesis are highlighted in yellow.
